# Office Buildings and Quacks

**Published:** 1903-02

**Authors:** 


### EDITORIAL NOTES AND COMMENTS.

---

The Business office of THE JOURNAL-RECORD is No. 64 Marietta Street.

The Editorial office is 318-319-320 The Prudential.

Address all Business communications to Dr. M. B. Hutchins, Mgr.

Make remittances payable to THE ATLANTA JOURNAL-RECORD OF MEDICINE.

On matters pertaining to the Editorial and Original Communications address Dr. Bernard Wolff, Atlanta.

Reprints of original articles will be furnished at cost price. Requests for the same should always be made *on the manuscript.*

We will present, post-paid, on request, to each contributor of an original article, twenty (20) marked copies of THE JOURNAL-RECORD containing such article.

---

### OFFICE BUILDINGS AND QUACKS.

SOME of the newspapers of this city have seen fit to criticize the managers of the large office buildings for excluding quacks and advertising physicians of all descriptions. The newspaper naturally takes side with the quacks for diplomatic and prudential reasons, as a not inconsiderable share of its income is derived from such patronage. That the managers are entirely justified in pursuing this policy, and that it is based on sound business principles, is easily demonstrated by a consideration of the local conditions. With but few exceptions the physicians of Atlanta have offices not in their residences, as is the usual custom elsewhere, but in the large buildings down-town. They make good tenants, pay rent promptly, and are more or less easy to please, and are permanent. Their presence in a building is a guaranty of its respectability and is an attraction. For these and other reasons the managers are disposed to defer to their prejudices and prepossessions. In the first rank of professional prejudice is the deep-rooted objection to propinquity with the medical prostitute—the quack. This objection is as rational as that of the house-owner who protests against the presence near his property of places of questionable resort and houses of ill-fame. He does not care to be put upon the defensive to explain wherein he differs from his neighbors upon whom suspicion rests. No manager can afford to harbor any person or persons who are offensive to his best tenants. It seems that the newspapers reason very much in the same way. Viewed in another light, quacks as a business proposition are not desirable for the manager of the large office building. They are essentially itinerant and roving in their habits. Their methods are notorious. They come to town heralded by the newspapers, rent an office by the week or month, install a stenographer, have some symptom blanks printed, and with these baits proceed to angle for suckers. It does not take very long to judge how much a town offers of this kind of spoil. If his advertising lures do not immediately attract, he concludes that there is nothing doing in that place, and leaving his impediments that are not readily portable or valuable as hostages for rent unpaid, moves to another locality with a celerity that is not altogether unsuggestive of flight. This is not the sort of tenant for whom the landlord yearns.

But the newspapers, arranging with the quack on the safe system of cash in advance for advertising, look on his methods with an indulgence that is not extended to them by others. So long as these messengers of civilization and bulwarks of freedom have such a very evident ax to grind, their criticisms of the business sagacity of the office building managers may be borne with considerable fortitude.

---

AT the recommendation of the Chief of the Bureau of Education, the post of specialist in criminology and cognate subjects has been done away with, and Mr. Arthur McDonald, the former incumbent, has been dropped. Mr. McDonald naturally has no desire to have his work thus summarily disposed of, and wishes to have the title of his position changed to director of laboratory to study criminal, defective and pauper classes, and to have this work done under the supervision of some other department. Mr. McDonald's studies in this field are already well and favorably known, and it is much to be hoped that the government will see fit to continue with his valuable services.

---

DR. EUGENE FOSTER, of Augusta, died of cancer of the stomach January 22. Dr. Foster was one of the best-known physicians in the State. At the time of his death he was the president of the Board of Trustees of the State Sanitarium at Milledgeville and also dean of the Medical Department of the State University. He made numerous contributions to medical literature, chiefly of an historical character. His death is a severe loss to the medical profession of Georgia.

---

THE Southern Section of the Laryngological, Otological and Rhinological Society met here on January 24. The attendance was very small, but the papers read were interesting and elicited free discussion. The visiting guests present from a distance were Drs. J. F. McKernon, Dench and McCaw, of New York. There seems to be urgent need of reform in the name of this association. It is a verbal procession and a tax upon the vocal organs that may mean "rhino" to the members but spells hardship to those who are not. Why not abbreviate the name of the society by using the initials L. O. R., and save some breath for the discussions?

---

THE engagement of Dr. Cyrus Strickler to Miss Williams has been announced.

